# Morphologic and molecular study of lung cancers associated with idiopathic pulmonary fibrosis and other pulmonary fibroses

**DOI:** 10.1186/s12931-017-0605-y

**Published:** 2017-06-15

**Authors:** Alice Guyard, Claire Danel, Nathalie Théou-Anton, Marie-Pierre Debray, Laure Gibault, Pierre Mordant, Yves Castier, Bruno Crestani, Gérard Zalcman, Hélène Blons, Aurélie Cazes

**Affiliations:** 1 Département de Pathologie, Hôpital Bichat-Claude Bernard, Assistance Publique-Hôpitaux de Paris, 46 rue Henri Huchard, 75018 Paris, France; 2 Département de Génétique, Hôpital Bichat-Claude Bernard, Assistance Publique-Hôpitaux de Paris, 46 rue Henri Huchard, 75018 Paris, France; 3 Service de Radiologie, Hôpital Bichat-Claude Bernard, Assistance Publique-Hôpitaux de Paris, 46 rue Henri Huchard, 75018 Paris, France; 4 Service de Pathologie, Hôpital Européen Georges Pompidou, Assistance Publique-Hôpitaux de Paris, 20 rue Leblanc, 75015 Paris, France; 5 Service de chirurgie vasculaire et thoracique, Hôpital Bichat-Claude Bernard, Assistance Publique-Hôpitaux de Paris, 46 rue Henri Huchard, 75018 Paris, France; 6 Service de Pneumologie A, Hôpital Bichat-Claude Bernard, Assistance Publique-Hôpitaux de Paris, 46 rue Henri Huchard, 75018 Paris, France; 7 INSERM U1152, DHU FIRE, Labex Inflamex, Université Paris- Diderot, Paris, France; 8 Service d’Oncologie Thoracique, CIC1425/CLIP2 Paris-Nord, Université Paris-Diderot, Hôpital Bichat-Claude Bernard, AP-HP, 46 rue Henri Huchard, 75018 Paris, France; 9 INSERM U830, Institut Curie, Paris, France; 10 Department of Biochemistry, Pharmacogenetic and Molecular Oncology Unit, Hôpital Européen Georges Pompidou, Assistance Publique - Hôpitaux de Paris, INSERM UMR-S1147, Université Sorbonne Paris Cité, Paris, France

**Keywords:** Idiopathic pulmonary fibrosis, Fibrosis-associated lung cancer, Next-generation sequencing

## Abstract

**Background:**

Primitive lung cancers developed on lung fibroses are both diagnostic and therapeutic challenges. Their incidence may increase with new more efficient lung fibrosis treatments. Our aim was to describe a cohort of lung cancers associated with idiopathic pulmonary fibrosis (IPF) and other lung fibrotic disorders (non-IPF), and to characterize their molecular alterations using immunohistochemistry and next-generation sequencing (NGS).

**Methods:**

Thirty-one cancer samples were collected from 2001 to 2016 in two French reference centers for pulmonary fibrosis - 18 for IPF group and 13 for non-IPF group. NGS was performed using an ampliseq panel to analyze hotspots and targeted regions in 22 cancer-associated genes. ALK, ROS1 and PD-L1 expressions were assessed by immunohistochemistry.

**Results:**

Squamous cell carcinoma was the most frequent histologic subtype in the IPF group (44%), adenocarcinoma was the most frequent subtype in the non-IPF group (62%). Forty-one mutations in 13 genes and one *EGFR* amplification were identified in 25 samples. Two samples had no mutation in the selected panel. Mutations were identified in *TP53* (*n* = 20), *MET* (*n* = 4), *BRAF* (*n* = 3), *FGFR3*, *PIK3CA*, *PTEN*, *STK11* (*n* = 2), *SMAD4, CTNNB1*, *DDR2*, *ERBB4*, *FBXW7* and *KRAS* (*n* = 1) genes. No ALK and ROS1 expressions were identified. PD-L1 was expressed in 10 cases (62%) with only one (6%) case >50%.

**Conclusions:**

This extensive characterization of lung fibrosis-associated cancers evidenced molecular alterations which could represent either potential therapeutic targets either clues to the pathophysiology of these particular tumors. These findings support the relevance of large molecular characterization of every lung fibrosis-associated cancer.

## Background

Idiopathic pulmonary fibrosis (IPF) is a chronic parenchymal lung disease of severe prognosis, with a median survival of about 3 years from diagnosis [1]. An increased incidence of lung cancer has been described in IPF patients, with a significantly adverse impact on survival [2–6]. IPF and lung cancer are both strongly associated with tobacco-smoking. Incidence of lung cancer is also increased in non-idiopathic pulmonary fibrosis suggesting a role for inflammation and fibrosis in the development of lung tumors [7]. Common pathogenic pathways and epigenetic alterations have been described in both IPF and cancer but specific molecular analysis of lung fibrosis-associated tumors has not been published so far [8].

Lung cancer in IPF patients is a therapeutic challenge as both surgery and radiotherapy are limited by lung dysfunction and are at high risk of respiratory exacerbation. Moreover chemotherapy can also be deleterious [5, 9]. However, over the past decade a better knowledge of lung cancer biology led to major changes in the management of lung cancer patients. Targeted therapies based on biomarkers have shown clinical success. Genetic alterations differ according to histologic subtypes. In adenocarcinoma (ADC), the most common cancer type, molecular characterization is now an established procedure before any therapeutic decision [10]. In squamous cell carcinoma (SCC), some targets have been identified but need to be validated [11]. Molecular alterations in oncogenes may confer constitutive activation and oncogenic addiction as for EGFR, the first target identified in lung ADC. More recently mutated BRAF and MET were also demonstrated to be addictive oncogenes. Finally, gene fusions, for instance *ALK* and *ROS1* are other molecular mechanisms leading to oncogene activation and are validated targets [12]. In parallel identification of the tumor immune-evasion mechanisms is the basis for innovative therapies, particularly targeting the PD-1/PD-L1 pathway. Although in need of standardization, PD-L1 expression as detected by immunohistochemistry may be a predictive biomarker of anti PD-1/PD-L1 drug’s efficacy [13].

The aim of this study was to describe a retrospective cohort of lung cancers developed on IPF and other pulmonary fibroses, and to search for molecular alterations that could either represent therapeutic targets or specific oncogenic pathways in these interstitial lung diseases (ILD).

## Methods

### Patients and tumors

Cases of lung fibrosis-associated lung cancer diagnosed between 2001 and 2016 were identified from clinical and pathological databases of Bichat-Claude Bernard and Georges Pompidou University hospitals (Paris, France), which are both “Competence Centers for rare pulmonary disorders”. Formalin-fixed and paraffin-embedded (FFPE) samples were retrieved from Pathology department archives. Two pathologists (AC, AG) reviewed all samples to confirm diagnoses of lung fibrosis and cancer. Cancers were classified according to the 2015 WHO Classification of Lung Tumors [14]. IPF and Idiopathic Interstitial Pneumonias were diagnosed according to American Thoracic Society–European Respiratory Society consensus criteria [1, 15]. The relationship between tumor and UIP lesions was assessed on 2 slides/tumor on surgical cases of the IPF group. This study was reviewed and approved by the CEERB Paris Nord ethics committee, under the number 16–007.

### Next-generation sequencing

The percentage of tumor cells was assessed by two pathologists (AC, AG), in a macrodissection area if required. DNA extraction from FFPE tissues was performed using Maxwell® 16 (Promega, Fitchburg, Wisconsin). DNA was quantified by Qubit® 2.0 Fluorometer (Qubit® dsDNA BR Assay kit-Life Technologies-Thermo Fisher Scientific, Saint Aubin, France). Sequencing libraries were prepared from tumor FFPE DNA using Ion AmpliSeq™ Colon and Lung Cancer Research Panel V2 (Life Technologies-Thermo Fisher Scientific). This panel targets over 500 hotspot mutations in 22 colon and lung cancer-associated genes: *AKT BRAF CTNNB1 EGFR ERBB2 ERBB4 FBXW7 FGFR1 FGFR2 FGFR3 KRAS MET NOTCH1 NRAS PIK3CA PTEN SMAD4 STK11 TP53 ALK DDR2 MAP2K1*. The multiplex barcoded libraries were generated with Ion AmpliSeq Library kit from 3-μL of DNA corresponding to 10–30ng. Using NGS data, we developed an algorithm that was used to test the presence of gene amplifications in this series. Amplifications were subsequently validated by qPCR.

MET mutations in the intronic region before the exon 14 were researched in 3 samples (P15, P24, P30) by HRM PCR (LC480, Roche, Basel, Switzerland) followed by Sanger sequencing (abi3130, Thermo Fisher Scientific, Waltham, Massachusetts, USA), using two amplicons of 200 and 212 bp around splice sites (at least 10 bp upstream and downstream).

Mutations were referred to the COSMIC database [16]. Pathogenicity prediction was studied using SIFT, Mutation Taster, PolyPhen and UMD pathogenicity prediction softwares [17–20].

### Immunohistochemistry

Immunohistochemistry was performed on fresh 5-μm sections from FFPE blocks on Leica BOND-MAX (Leica Biosystems, Buffalo Grove, IL) automated staining system. Briefly, slides were deparaffinized and subjected to antigen retrieval in a pH = 9 buffer. Primary antibodies (ALK – clone 5A4 – Abcam, Cambridge, UK, 1:50 dilution; ROS-1 – clone D4D6 – Genemed Biotechnologies, San Francisco, CA, 1:100 dilution; PD-L1 – clone E1L3N – Cell Signaling Technology, Danvers, MA, 1:400 dilution) were incubated for 60, 60 and 20 min respectively. Revelation was performed with Leica BOND-MAX detection kits. ALK and ROS1 results were interpreted as positive or negative. PD-L1 result was expressed as the percentage of stained tumor cells.

### Statistical analysis

Continuous variables are described by their mean and SD, and compared by use of Student’s *t*-test. Categorical variables are described by percentages and compared by Fisher’s exact test. Statistical analysis used Prism 5 (GraphPad Software, La Jolla, CA). *P* < 0.05 was considered statistically significant.

## Results

### Patients

Thirty-one tumor samples were collected from 30 patients (Table 1). Eighteen were collected from patients diagnosed with IPF and 13 from patients suffering from other lung fibrotic disorders: connective tissue disease-associated interstitial lung disease (CTD-ILD) *n* = 6, idiopathic non-specific interstitial pneumonia *n* = 2, pneumoconiosis *n* = 4, drug-induced lung fibrosis *n* = 1.

**Table 1:** Clinical features

Patient	Gender	Age (years)	Tobacco (P-Y)	Disease	CT-scan	Cancer type	Cancer location	Sampling site and mode
Idiopathic pulmonary fibrosis								
P1	M	86	<5	IPF	UIP	SCC	peripheral	Lung, biopsy
P2	F	63	40	IPF	UIP	SCC	peripheral	Lung, biopsy
P3	M	60	NP	IPF	UIP	SCC	peripheral	Lung, surg. resec.
P4	M	55	40	IPF	UIP	SCC	peripheral	Lung, surg. resec.
P5	M	41	30	IPF	UIP	SCC	peripheral	Lung, biopsy
P6	M	69	45	IPF	UIP	SCC	proximal	LN, EBUS
P7	M	75	30	IPF	UIP	SCC	peripheral	Lung, surg. resec.
P8	M	66	yes (NS)	likely IPF	UIP	SCC	peripheral	Lung, surg. resec.
P9	M	68	20	IPF	UIP	ADC	peripheral	Lung, biopsy
P10	F	56	35	IPF	UIP	ADC	peripheral	Lung, biopsy
P3	M	61	NS	IPF	UIP	ADC	peripheral	Lung, autopsy
P11	M	62	0	IPF	UIP	ADC	peripheral	Pleural liquid
P12	M	58	50	IPF	UIP	ADC	peripheral	Lung, surg. resec.
P13	M	64	40	likely IPF	UIP	ADC	peripheral	Lung, surg. resec.
P14	M	73	55	IPF	UIP	ADS	proximal	Lung, surg. resec.
P15	M	67	10	IPF	UIP	ADS	peripheral	Lung, surg. resec.
P16	M	57	60	likely IPF	UIP	LCNEC	peripheral	LN, biopsy
P30	M	51	30	IPF	UIP	SmCC	peripheral	Lung, biopsy
Connective Tissue Disease-Interstitial Lung Disease								
P18	M	57	40	RA	NSIP	SCC	proximal	Lung, surg. resec.
P20	F	55	10	RA	UIP	ADC	peripheral	Lung, surg. resec.
P21	M	69	100	RA	UIP	ADC	peripheral	Lung, surg. resec.
P24	M	62	40	RA	NSIP	ADS	peripheral	Lung, surg. resec.
P23	M	66	30	antisynthetase sd	NSIP	ADC	peripheral	LN, biopsy
P22	F	59	0	scleroderma	UIP	ADC	peripheral	Lung, surg. resec.
Non-specific interstitial pneumonia								
P25	M	69	70	NSIP	NSIP	ADC	peripheral	Lung, surg. resec.
P26	F	54	60	NSIP	NSIP	ADC	peripheral	Lung, surg. resec.
Pneumoconiosis								
P17	M	64	50	pneumoconiosis	Em-UIP	SCC	peripheral	Lung, surg. resec.
P27	M	59	17	asbestosis	UIP	ADC	peripheral	Lung, biopsy
P19	M	58	yes (NS)	Iikely asbestosis	UIP	SCC	peripheral	Lung, biopsy
P29	M	73	50	asbestosis	Em-UIP	SmCC	peripheral	Lung, biopsy
Drug-induced lung fibrosis								
P28	M	87	60	NC (amiodarone?)	ILD	ADC	peripheral	Lung, biopsy

*ADC* adenocarcinoma, *ADS* adenosquamous carcinoma, *EBUS* endobronchial ultrasound, *Em* emphysema, *IPF* idiopathic pulmonary fibrosis, *LCNEC* large cell neuro-endocrine carcinoma, *LN* lymph node, *NS* not specified, *NSIP* non-specific interstitial pneumonia, *P-Y* pack-years, *RA* rheumatoid arthritis, *SCC* squamous cell carcinoma, *SmCC* small cell carcinoma, *surg. resec* surgical resection, *UIP* usual interstitial pneumonia

Men predominate in both groups (89% in IPF group and 77% in non-IPF group, *n* = 0.62). No difference was observed in age (63 +/− 9.9 vs 64 +/− 9.1, *p* = 0.75) and tobacco use (never smoker: 5.5% vs 7.6%, *p* = 0.74).

Samples were collected from surgical resection (n = 16), lung core biopsy (*n* = 10), lymph node core biopsy/cytology (*n* = 3), autopsy (*n* = 1) and pleural fluid (*n* = 1). Age of FFPE material ranged from 0 to 13 years (mean = 3.5 +/− 3.3).

### Pathologic characterization

Pathologic characterization is summarized in Table 2. In the IPF group, histologic subtypes were SCC (*n* = 8, 44%), ADC (*n* = 6, 33%), adenosquamous carcinoma (ADS) (*n* = 2, 11%), small cell carcinoma (SmCC) (*n* = 1, 6%) and large cell neuro-endocrine carcinoma (LCNEC) (*n* = 1, 6%). In the non-IPF group, histologic subtypes were ADC (*n* = 8, 62%), SCC (*n* = 3, 23%), ADS (*n* = 1, 8%) and SmCC (*n* = 1, 8%).

**Table 2:** Pathological features

Patient	Cancer type	Cancer differenciation	Diagnostic immunohistochemistry (IHC)			Therapeutic IHC		
			TTF1	p40/p63	others	ALK	ROS1	PDL1
Idiopathic pulmonary fibrosis								
P1	SCC	keratinizing	/	/		/	/	/
P2	SCC	nonkeratinizing	TTF1-	p40+		/	/	/
P3	SCC	basaloid,	/	p63+	CK7-	/	/	<1%
		keratinizing						
P4	SCC	keratinizing	TTF1-	p40+		/	/	5%
P5	SCC	nonkeratinizing	TTF1-	p63+	NapsinA- CK5/6+	/	/	/
P6	SCC	keratinizing	TTF1-	p63+		/	/	/
P7	SCC	keratinizing	TTF1-	p40+		/	/	10%
P8	SCC	nonkeratinizing	TTF1-	p40+		/	/	0%
P9	ADC	acinar	TTF1+		CK7+	neg	/	/
P10	ADC	acinar	TTF1-	p63-		/	/	/
P3	ADC	solid	TTF1+	p63-		neg	neg	<1%
P11	ADC	NS	TTF1-	p63-	NapsinA+	/	/	/
P12	ADC	mucinous	TTF1-	/	CK7+ CK20+	neg	/	/
P13	ADC	acinar	TTF1+	p40-	CK7+ CD56-	neg	neg	1%
P14	ADS	acinar	TTF1-	p40+	CK7+	neg	neg	20%
P15	ADS	papillary	TTF1+	p40+		neg	neg	15%
P16	LCNEC	/	TTF1-	/	chromoA+ CD56+	/	/	/
					synapto + CK5/6-			
P30	SmCC	/	TTF1+	/	chromoA+ CD56+	/	/	/
					synapto+			
Connective Tissue Disease-Interstitial Lung Disease								
P18	SCC	keratinizing	TTF1-	p40+		/	/	40%
P20	ADC	papillary	TTF1+	/		neg	neg	<1%
P21	ADC	solid	TTF1+	p40-		neg	neg	70%
P24	ADS	solid	TTF1+	p40+		neg	neg	10%
P23	ADC	solid	TTF1+	p63-	NapsinA+	/	/	/
P22	ADC	acinar	TTF1+	/	CK7+	neg	neg	0%
Non-specific interstitial pneumonia								
P25	ADC	acinar	TTF1+	p40+		neg	neg	<1%
P26	ADC	papillary	TTF1+	/		neg	neg	1%
Pneumoconiosis								
P17	SCC	keratinizing	TTF1-	p40+		/	/	1%
P27	ADC	solid	TTF1+	p40+		/	/	/
P19	SCC	nonkeratinizing	TTF1-	p63+	CK5/6+	/	/	/
P29	SmCC	/	TTF1-	/	CD56+	/	/	/
Drug-induced lung fibrosis								
P28	ADC	acinar	TTF1+	/	NapsinA+	/	/	/

*ADC* adenocarcinoma, *ADS* adenosquamous carcinoma, *LCNEC* large cell neuro-endocrine carcinoma, *SCC* squamous cell carcinoma, *SmCC* small cell carcinoma

**Fig. 1:**
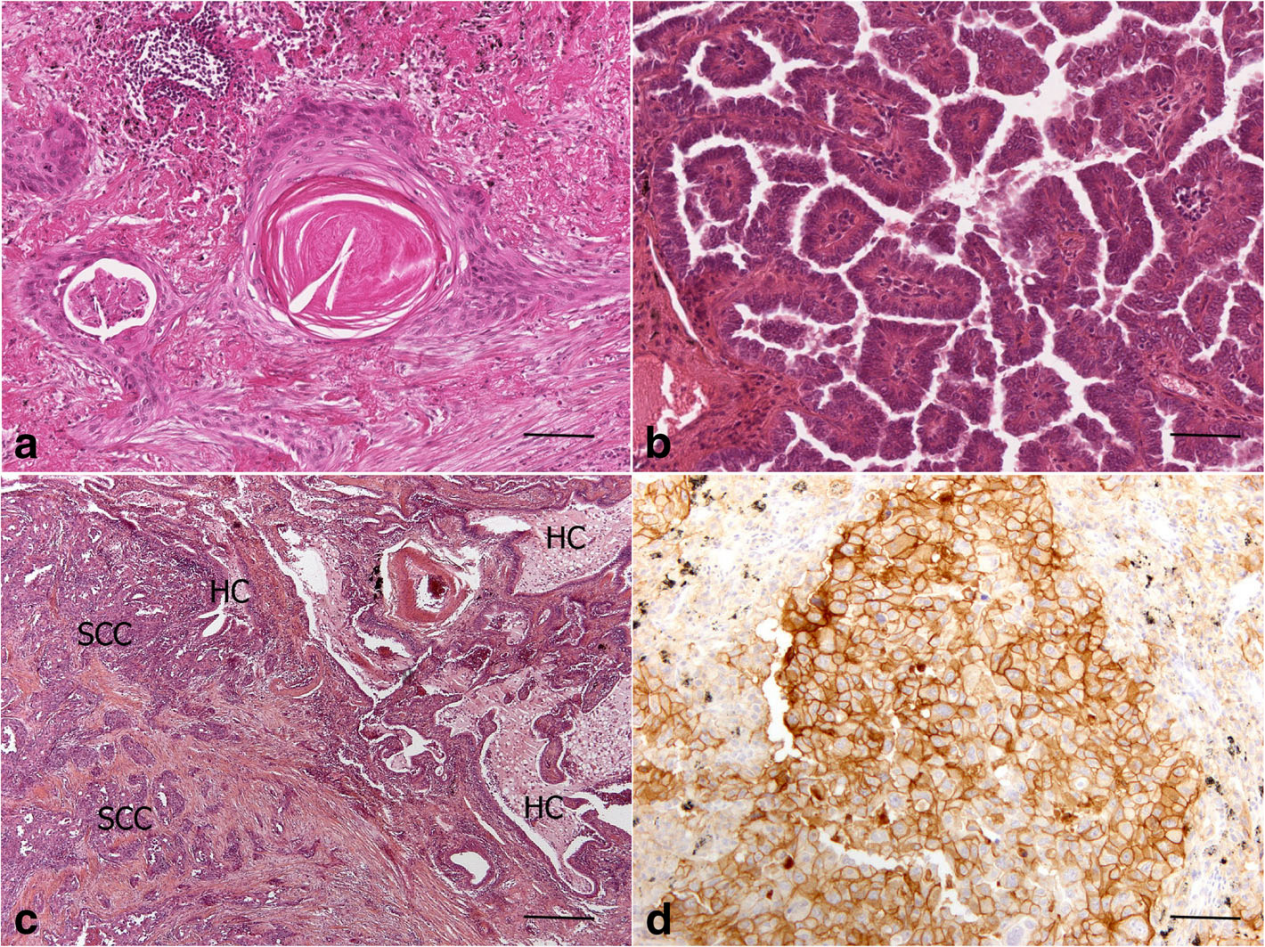
Pathological and immunohistochemical characteristics of lung tumors. **a** Keratinising squamous cell carcinoma (P4, HES, x20 objective, scale bar:100μm) (**b**) Papillary adenocarcinoma (P26, HES, x20 objective, scale bar:100μm) (**c**) Peripheral squamous cell carcinoma developed in honeycomb lung (HES, x5 objective, scale bar:500μm) (**d**) Positive PD-L1 staining (P21, anti-PD-L1 immunohistochemistry, x20 objective, scale bar:100μm)

Six of the 11 SCC (55%) were keratinizing and one was basaloid (Fig. 1a). In ADC, acinar (*n* = 6, 43%) and solid (*n* = 4, 29%) were the most frequent subtypes, both observed in IPF and non-IPF groups. Papillary (*n* = 2, 14%) subtype was observed in the non-IPF group and mucinous (*n* = 1, 7%) subtype in the IPF group (Fig. 1b). A high proportion of tumors were peripheral in both groups: 16/18 (89%) in IPF group and 12/13 (92%) in non-IPF group. In the IPF group, 7/9 surgically removed tumors were developed in close contact with peripheral honeycomb regions (Fig. 1c). Two out of 9 were in contact with emphysema lesions.

### Immunohistochemistry

PD-L1 expression was assessed in all surgical resections and in the autopsy specimen, corresponding to 16 cases (6 SCC, 7 ADC and 3 ADS). Among them, 6 had less than 1% of stained tumor cells, 3 had 1% to <5%, 6 had 5% to <50% and one ADC had more than 50% of stained tumor cells. Overall, 10 tumors (62%) should be considered as expressing tumor cell membrane PD-L1 antigen in more than 1% of cells (Table 2 and Fig. 1d), and one (6%) with a high level of expression.

ALK and ROS1 expression was assessed in all ADC from surgical resections and autopsy specimen (*n* = 10). For two other patients, ALK expression was assessed during the patient management (P9 and P12). In all tested cases, ALK and ROS1 were negative.

### Next-generation sequencing

In 27/31 samples (87%), DNA quality was sufficient for proper analysis. The mean coverage was 10,646 (median 5,687, range from 247.8 to 34,874).

NGS results are presented in Tables 3 and 4. One or more mutations were found in 25/27 samples (93%). Eleven samples (41%) had one mutation, eight (30%) two mutations, five (19%) three mutations, and one (4%) presented an *EGFR* gene amplification.

**Table 3:** NGS results, *TP53* mutations

Gene		Mutation		COSMIC reference	Pathogenicity prediction	Patient	Allelic frequency	% tum cells	Lung disease	Cancer
*TP53*	Chr17:g.7579383T > G	c.304A > C	p.Thr102Pro	/	benign	P09	11.0	NS	IPF	ADC
	Chr17:g.7578461C > A	c.469G > T	p.Val157Phe	COSM10670	pathogenic	P18	54.0	70	CTD-ILD	SCC
	Chr17:g.7578457C > A	c.473G > T	p.Arg158Leu	COSM10714	pathogenic	P20	27.8	40	CTD-ILD	ADC
	Chr17:g.7578454G > A	c.476C > T	p.Ala159Val	COSM11148	pathogenic	P15	44	NS	IPF	ADS
	Chr17:g.7578406C > T	c.524G > A	p.Arg175His	COSM10648	pathogenic	P21	42.2	70	CTD-ILD	ADC
	Chr17:g.7578388C > G	c.542G > C	p.Arg181Pro	COSM45046	pathogenic	P09	22.1	NS	IPF	ADC
	Chr17:g.7578272G > T	c.577C > A	p.His193Asn	COSM43935	pathogenic	P03-ADC	59.3	70	IPF	ADC
	Chr17:g.7578272G > A	c.577C > T	p.His193Tyr	COSM10672	pathogenic	P01	22.4	50	IPF	SCC
	Chr17:g.7577574T > C	c.707A > G	p.Tyr236Cys	COSM10731	pathogenic	P30	84.0	70	IPF	SmCC
	Chr17:g.7577559G > A	c.722C > T	p.Ser241Phe	COSM10812	pathogenic	P27	16.4	20	pneumoconiosis	ADC
	Chr17:g.7577559G > A	c.722C > T	p.Ser241Phe	COSM10812	pathogenic	P29	84.7	>50	pneumoconiosis	SmCC
	Chr17:g.7577539G > A	c.742C > T	p.Arg248Trp	COSM10656	pathogenic	P11	42.8	70	IPF	ADC
	Chr17:g.7577535C > A	c.746G > T	p.Arg249Met	COSM43871	pathogenic	P08	28.9	40	IPF	SCC
	Chr17:g.7577535C > A	c.746G > T	p.Arg249Met	COSM43871	pathogenic	P24	61.0	70	CTD-ILD	ADS
	Chr17:g.7577120C > A	c.818G > T	p.Arg273Leu	COSM10779	pathogenic	P16	68.1	40	IPF	LCNEC
	Chr17:g.7577115dup	c.823dup	p.Cys275Leufs*31	/	pathogenic	P04	16.2	25	IPF	SCC
	Chr17:g.7577108C > A	c.830G > T	p.Cys277Phe	COSM10749	pathogenic	P02	42.5	40	IPF	SCC
	Chr17:g.7577096_7577099del	c.839_842del	p.Arg280Thrfs*64	/	pathogenic	P05	63.6	30	IPF	SCC
	Chr17:g.7577046C > A	c.892G > T	p.Glu298*	COSM10710	pathogenic	P19	65.1	40	pneumoconiosis	SCC
	Chr17:g.7573976T > A	c.1051A > T	p.Lys351*	COSM1522202	pathogenic	P17	61.1	90	pneumoconiosis	SCC

*ADC* adenocarcinoma, *ADS* adenosquamous carcinoma, *CTD-ILD* connective tissue disease associated-interstitial lung disease, *IPF* idiopathic pulmonary fibrosis, *LCNEC* large cell neuro-endocrine carcinoma, *NSIP* non-specific interstitial pneumonia, *SCC* squamous cell carcinoma, *SmCC* small cell carcinoma

**Table 4:** NGS results, other mutations

Gene		Mutation		COSMIC reference	Pathogenicity prediction	Patient	Allelic frequency	% tum cells	Lung disease	Cancer
*MET*	Chr7:g.116340214G > A	c.1076G > A	p.Arg359Gln	COSM1286164	probably benign	P01	49.4	50	IPF	SCC
	Chr7:g.116411867G > A	c.2942–36G > A				P15		NS	IPF	ADS
	Chr7:g.116411923C > T	c.2962C > T	p.Arg988Cys	COSM1666978	unknown	P05	33.9	30	IPF	SCC
	Chr7:g.116411992A > G	c.2977A > G	p.Thr1011Ala	/	unknown	P22	27.6	50	CTD-ILD	ADC
*BRAF*	Chr7:g.140481402C > G	c.1406G > C	p.Gly469Ala	COSM460	pathogenic	P17	71.8	90	pneumoconiosis	SCC
	Chr7:g.140481402C > G	c.1406G > C	p.Gly469Ala	COSM460	pathogenic	P28	46.9	70	drug-induced LF	ADC
	Chr7:g.140453134T > C	c.1801A > G	p.Lys601Glu	COSM478	pathogenic	P20	27.6	40	CTD-ILD	ADC
*PIK3CA*	Chr3:g.178936082G > A	c.1624G > A	p.Glu542Lys	COSM760	pathogenic	P28	64.2	70	drug-induced LF	ADC
	Chr3:g.178936082G > A	c.1624G > A	p.Glu542Lys	COSM760	pathogenic	P15	48	NS	IPF	ADS
	Chr3.g.178938847A > T	c.2089A > T	p.Met697Leu	/	unknown	P25	8.5	50	NSIP	ADC
*FGFR3*	Chr4:g.1806149G > C	c.1168G > C	p.Val390Leu	/	unknown	P25	9.6	50	NSIP	ADC
	Chr4:g.1807891G > C	c.1950G > C	p.Lys650Asn	COSM3993568	pathogenic	P29	16.3	>50	pneumoconiosis	SmCC
*PTEN*	Chr10:g.89720729del	c.880del	p.Ser294Valfs*13	/	pathogenic	P02	20.1	40	IPF	SCC
	Chr10:g.89720852C > T	c.1003C > T	p.Arg335*	COSM5151	pathogenic	P02	34.5	40	IPF	SCC
*STK11*	Chr19:g.1221249del	c.772del	p.Asp258Thrfs*29	/	pathogenic	P06	93.6	50	IPF	SCC
	Chr19:g.1223125C > G	c.1062C > G	p.Phe354Leu	COSM21360	benign	P20	49.1	40	CTD-ILD	ADC
*SMAD4*	Chr18:g.48591865C > G	c.1028C > G	p.Ser343*	COSM14111	pathogenic	P05	17.7	30	IPF	SCC
*CTNNB1*	Chr3:g.41266113C > G	c.110C > G	p.Ser37Cys	COSM5679	pathogenic	P26	34.4	50	NSIP	ADC
*DDR2*	Chr1:g.162729689T > A	c.775T > A	p.Trp259Arg	/	pathogenic	P24	33.0	70	CTD-ILD	ADS
*ERBB4*	Chr2:g.212576809C > A	c.1090G > T	p.Gly364Trp	/	pathogenic	P25	18.2	50	NSIP	ADC
*FBXW7*	Chr4:g.153249370G > A	c.1408C > T	p.His470Tyr	/	probably pathogenic	P06	29.5	50	IPF	SCC
*KRAS*	Chr12:g.25398285C > A	c.34G > T	p.Gly12Cys	COSM516	pathogenic	P26	35.1	50	NSIP	ADC
*EGFR* amplification (6.5 copies)							P10	10	IPF	ADC

**Fig. 2:**
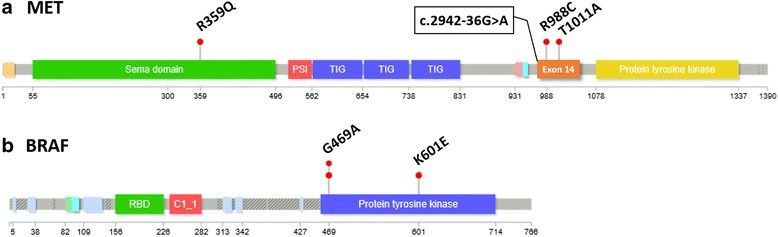
*MET* and *BRAF* mutations. **a** Three exonic mutations: p.Arg359Gln, p.Arg988Cys and p.Thr1011Ala and one intronic mutation: c.2942-36G > A were detected within *MET* gene. **b** Two p.Gly469Ala and one p.Lys601Glu *BRAF* mutations were detected. Diagrams were made with the Lollipops software (https://github.com/pbnjay/lollipops)

Forty-four molecular alterations were identified in 14 genes. Twenty *TP53* mutations were detected (Table 3). Nine molecular alterations were found in four genes coding for tyrosine kinase receptors: point mutations in *MET* (4) (Fig. 2a), *FGFR3* (2), *ERBB4* (1) and *DDR2* (1) and one *EGFR* amplification. Seven mutations were described in the PI3K pathway, involving *PIK3CA* (3), *PTEN* (2) and *STK11* (2) genes. Four mutations involving the MAPK pathway were identified in *BRAF* (3) (Fig. 2b) and *KRAS* (1) (Table 4). Single *TP53* mutations were observed in 11 patients. Single mutation in another oncogenic gene was found in one case (*MET* gene for P22). Multiple oncogenic activations were found in 12 patients.

Mutations classified by histologic subtype are in SCC: *TP53* (*n* = 8, 80%), *MET* (*n* = 2, 20%), *BRAF*, *PTEN*, *SMAD4*, *STK11* and *FBXW7* (*n* = 1, 10%); in ADC: *TP53* (*n* = 6, 50%), *BRAF* and *PIK3CA* (n = 2, 17%), *MET*, *FGFR3*, *STK11*, *CTNNB1*, *ERBB4*, *KRAS* and *EGFR* amplification (*n* = 1, 8%). Two mutations of *TP53* and one mutation of *PIK3CA, MET* and *DDR2* were found in the 2 ADS.

Mutations analysed according to parenchymal disease subtype are, in IPF group: *TP53* (*n* = 11, 73%), *MET* (*n* = 3, 20%), *PTEN*, *SMAD4*, *FBXW7*, *STK11, PIK3CA* and *EGFR* amplification (*n* = 1, 7%); in non-IPF group: *TP53* (*n* = 8, 67%), *BRAF* (*n* = 3, 25%), *FGFR3* and *PIK3CA* (*n* = 2, 17%), *STK11*, *DDR2*, *MET*, *KRAS*, *ERBB4* and *CTNNB1* (*n* = 1, 8%).

## Discussion

The aim of this study was to describe a cohort of lung cancers developed on IPF and other pulmonary fibroses, and to characterize their molecular alterations. SCC was the most frequent histologic subtype in our IPF group, as mostly reported in previous studies encompassing a large period of time [3, 21]. This squamous histology could suggest specific oncogenic events in the IPF micro-environment where peripheral honeycomb-associated squamous metaplasia and dysplasia has been reported [22]. In contrast, ADC was the most frequent subtype in the heterogeneous non-IPF group, like in the general population. Acinar subtype was the most frequent ADC subtype in our cohort (43%), and invasive mucinous subtype was rare (7%), as reported in a 89 idiopathic interstitial pneumonia-associated ADC cases recent Japanese series (35.95% and 11.24% respectively), described by Kojima [23]. In another recent Japanese series on 44 UIP-associated ADC reported by Masai, invasive mucinous subtype was predominant (29.5% of ADC) [6].

Among the genes assessed in the NGS panel, we detected 43 mutations in 13 genes and an *EGFR* gene amplification in 25 samples.

We detected *TP53* mutations in 8 SCC (80% of SCC) and 6 ADC (50% of ADC), with the same frequency as reported in the literature [11]. We also detected *TP53* mutations in all other cancer subtypes. Allelic ratios suggest a loss of the second *TP53* allele, as usually in cancers [24]. Detected mutations occurring in the DNA binding domain (from codon 125 to 300), especially the hotspot codons in CpG sites, are similar to those already described, according to the COSMIC public database [16]. More than one third are G > T transversions, in accordance with the high proportion of smokers [25]. Thus a specific carcinogenesis process differing from tobacco smoke DNA signature and linked to chronic lung inflammation could not be inferred from this molecular analysis.

Four *MET* mutations were detected in our cohort: p.Arg359Gln and p.Arg988Cys in SCC (20%), p.Thr1011Ala in one ADC (8%) and c.2942-36G > A in one ADS. In the literature, *MET* mutations are reported in 2% to 7% of lung ADC and in 1% of lung SCC [12]. Codon 359 is located within the SEMA domain, involved in binding with the MET-specific ligand HGF. Codons 988 and 1011 are located in the exon 14, and c.2942-36G > A in the intronic region before the exon 14, required for negative regulation of MET. Mutations involving exon 14 splicing site have been described in lung ADC, they mostly result in exon 14 skipping and ultimately in MET protein stabilization [12, 26]. Case reports have demonstrated responses to MET-inhibitors in ADC patients with *MET*ex14 alterations [26]. *MET*ex14 mutations were, so far, not reported in lung SCC. These three exonic mutations have been described as rare polymorphisms. However their functional impact remains unclear as discordant results are obtained with pathogenicity prediction softwares. For instance p.Arg988Cys, although described as a germline polymorphism (rs34589476), has been reported in numerous lung cancers, and its pathogenic role remains elusive, in vitro data supporting functional consequences [27, 28]. Interestingly, in our cohort, three MET mutations occurred in IPF and 1 in CTD-ILD with an UIP pattern on CT-scan. Whether these variants represent true oncogenic drivers or significant polymorphisms in the fibrotic process, this could suggest a specific pathway in IPF/UIP lung with activation of the HGF/MET axis [29]. The search for MET mutations in non-tumoral IPF lung would be mandatory to test these hypotheses. Of note, we looked for mutations in flanking introns of exon 14 in only three cases. Thus we cannot exclude the possibility of more MET mutations. Whether such alterations could be targetable would deserve specific clinical trials.

A p.Trp259Arg *DDR2* mutation was observed in an ADS. In the literature, *DDR2* mutations are found in 4% of lung SCC and in 1% of lung ADC, without hotspot mutations. Clinical response to dasatinib was reported in rare case-reports of patients with lung SCC [30].

No mutation of *EGFR* was observed in our cohort, although reported in 10–15% of lung ADC [12]. This result, in addition to the absence of ALK and ROS1 rearrangement, is consistent with the predominance of male smokers in our cohort. Three recent Japanese studies also described a significantly lower *EGFR* mutation frequency in ILD/IPF patients [5, 6, 23].

Mutations involving the MAP kinase pathway are frequent in ADC [12]. We described a p.Gly469Ala *BRAF* mutation in a SCC (10% of SCC), a p.Lys601Glu and a p.Gly469Ala *BRAF* mutation in 2 ADC (17% of ADK). In the literature, *BRAF* mutations are reported in about 4% of lung SCC and in 10% of lung ADC [11, 12]. *BRAF* mutations p.Lys601Glu and p.Gly469Ala have already been described in lung ADC. Non-V600E mutations are usual, representing about half of *BRAF* mutations [31]. Conversely, p.Gly469Ala has never been described in lung SCC. Both are activating *BRAF* mutations. BRAF and MEK inhibitors can target p.V600E *BRAF* mutations [31, 32]. Response rates for lung cancer patients with non-V600 mutations are unknown. Only one ADC was *KRAS* mutated (representing 8% of adenocarcinomas) whereas *KRAS* mutations are reported in more than 30% of lung ADC [12], especially in smokers. While the absence of *EGFR* mutation could be explained by the high smoking rate in our population, the low incidence of *KRAS* mutations could suggest the implication of other oncogenic drivers possibly related to the chronic lung injury during the fibrotic process. Interestingly the recent series described by Masai et al*.* included frequent invasive mucinous ADC (29,5%), associated with numerous *KRAS* mutations (30,2%) [6]. This could suggest carcinogenesis differences linked to ethnicity or be the reflect of our limited number of patients. However these results were not confirmed by Kojima et al. who reported a low rate of invasive mucinous subtype (11,24%) and no difference of *KRAS* mutation rate between non-UIP-ADC and UIP-ADC [23].

One *SMAD4* mutation was found in one SCC-IPF tumors. *SMAD4* is a tumor-suppressive gene that can cause cell cycle arrest and apoptosis of epithelial cells, and is inactivated by mutation in over half of pancreatic cancers [33]. It acts as a central mediator in the transforming growth factor-β (TGF-β) signalling pathway. *SMAD4* mutations are uncommon in lung cancer, according to COSMIC database. However this signalling pathway, targeted by TGF-beta, could be of particular relevance in a lung fibrosis context. pSer343* predicted as pathogenic is located in the MH2 region which is implicated in the oligomerization of the protein which is essential for TGFbeta signalling [34].

A p.Ser37Cys *CTNNB1* mutation was detected in an ADC (8%). The codon 37 is a known hotspot mutation, implied in the constitutive activation of the Wnt signalling pathway, and the p.Ser37Cys mutation has been reported in lung ADC [35]. Mutated beta-catenin (CTNNB1) accumulation is followed by translocation to the nucleus and action in a transcriptional complex involving other transcriptional regulators like YAP1 to modulate apoptosis, proliferation or epithelial-mesenchymal transition [36].

A p.His470Tyr *FBXW7* mutation was detected in a SCC (10%). *FBXW7* mutations are uncommon in lung cancer, according to COSMIC. *FBXW7* is implicated in proteasome degradation of specific substrates and control tumorigenesis, acting on cell cycle, differentiation and apoptosis [37]. It is also involved in epithelial-to-mesenchymal transition by controlling mTOR pathway [38]. A p.Arg465His *FBXW7* mutation was reported in a lung ADC; the patient benefited from the mTOR inhibitor temsirolimus [39].

Besides molecular targeted therapies, immunotherapy using checkpoint inhibitors is a new efficient therapy against lung cancer. PD-L1 is an immune-checkpoint protein, interacting with its ligand PD-1 expressed by T-cells, used by the tumoral cell to escape the antitumor immune response. Several drugs target the PD-1/PD-L1 interaction. An association between therapeutic response and PD-L1 expression on tumor cells has been described, although it is not a binary predictive marker and the PD-L1 assays need further standardization and validation [13]. PD-L1 expression was assessed in 16 surgical cases in the current work. All ADC but one had less than 5% of stained tumor cells, which, in addition to the pulmonary adverse effects of these molecules, may not plead for a first-line use of immunotherapy in these patients. This has to be investigated in larger series. As far as SCC are concerned PD-L1 expression seems to be less correlated to efficacy, at least in second-line of treatment [40].

## Conclusion

We report here for the first time, to our best knowledge, an extensive pathological and molecular analysis of lung fibrosis-associated lung cancers. We found potentially actionable alterations in *MET, FGFR3, ERBB4, DDR2, EGFR, BRAF, PI3KCA* genes in various histologic subtypes. While most detected mutations are likely tobacco-associated *TP53* mutations, others may suggest alternative oncogenesis mechanisms: notably we found *MET*, *FGFR3, SMAD4* and *CTNNB1* mutations, all genes that could potentially be involved in the lung fibrosis process, either participating to epithelial-mesenchymal transition or the regulation or TGFβ pathway. Conversely, the low prevalence of *KRAS* mutations, contrasting with the high percentage of smokers, also supports a role for endogenous carcinogenic mechanisms linked to lung fibrosis. Although limited by the size of the cohort, our series shows the feasibility of such systematic molecular characterization, for both therapeutic and pathophysiological purposes. The high mortality of fibrotic lung diseases implies that cancer remains a rare complication since possibly occurring late in the course of fibrosis. Two recently approved drugs, pirfenidone and nintedanib, have been shown to slow IPF progression [41], and are expected to extend survival. If confirmed this may lead to an increase of challenging cancer cases and encourage to perform a large molecular characterization to every lung fibrosis-associated cancer.

## Abbreviations

ADC: Adenocarcinoma
ADS: Adenosquamous carcinoma
CTD-ILD: Connective tissue disease-associated interstitial lung disease
FFPE: Formalin-fixed and paraffin-embedded
IPF: Idiopathic pulmonary fibrosis
LCNEC: Large cell neuro-endocrine carcinoma
NGS: Next-generation sequencing
SCC: Squamous cell carcinoma
SmCC: Small cell carcinoma
